# Evolutionary assembly of the plant terrestrialization toolkit from protein domains

**DOI:** 10.1098/rspb.2024.0985

**Published:** 2024-07-31

**Authors:** Amra Dhabalia Ashok, Sophie de Vries, Tatyana Darienko, Iker Irisarri, Jan de Vries

**Affiliations:** ^1^ Department of Applied Bioinformatics, University of Goettingen, Institute for Microbiology and Genetics, Goldschmidtstr. 1, Goettingen 37077, Germany; ^2^ University of Goettingen, Campus Institute Data Science (CIDAS), Goldschmidstr. 1, Goettingen 37077, Germany; ^3^ Section Phylogenomics, Centre for Molecular biodiversity Research, Leibniz Institute for the Analysis of Biodiversity Change (LIB), Museum of Nature Hamburg, Martin-Luther-King-Platz 3, Hamburg 20146, Germany; ^4^ Department of Applied Bioinformatics, University of Goettingen, Goettingen Center for Molecular Biosciences (GZMB), Goldschmidtstr. 1, Goettingen 37077, Germany

**Keywords:** streptophyte algae, mosaic evolution, reductive evolution, plant terrestrialization, protein domains, plant evolution

## Abstract

Land plants (embryophytes) came about in a momentous evolutionary singularity: plant terrestrialization. This event marks not only the conquest of land by plants but also the massive radiation of embryophytes into a diverse array of novel forms and functions. The unique suite of traits present in the earliest land plants is thought to have been ushered in by a burst in genomic novelty. Here, we asked the question of how these bursts were possible. For this, we explored: (i) the initial emergence and (ii) the reshuffling of domains to give rise to hallmark environmental response genes of land plants. We pinpoint that a quarter of the embryophytic genes for stress physiology are specific to the lineage, yet a significant portion of this novelty arises not de novo but from reshuffling and recombining of pre-existing domains. Our data suggest that novel combinations of old genomic substrate shaped the plant terrestrialization toolkit, including hallmark processes in signalling, biotic interactions and specialized metabolism.

## 1. Introduction

**Figure 1.**
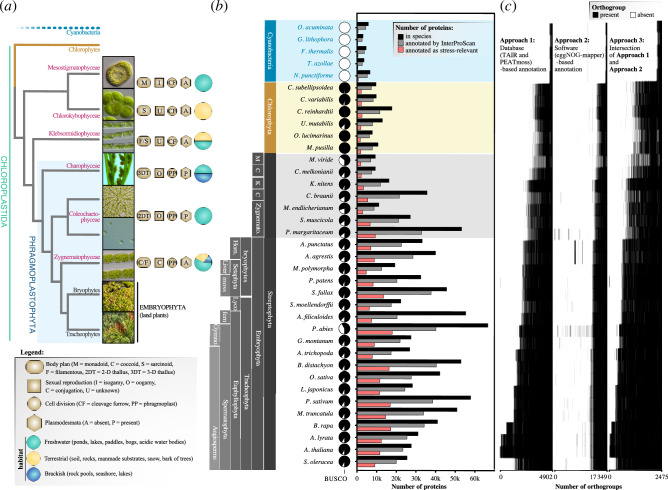
Stress-relevant orthogroups across the green lineage. (*a*) Summary of the phylogeny, morphology, life history and ecology in cyanobacteria (blue) and the green lineage that includes chlorophyte algae (ochre) and the focus of this study: streptophytes, encompassing streptophyte algae (purple) and land plants (grey). (*b*) A phylodiverse set of land plants and algae assembled from the predicted proteomes of 37 species. Pie charts show BUSCO proteome completeness. Bar graphs show the total number of proteins per proteome (black), the subset annotated by InterProScan [13] (grey) and the subset that were stress-relevant from approach 3 in figure 1*b* (red). (*c*) Number of stress-annotated orthogroups (homologous protein group) were compiled using three approaches, building on: (1) the databases TAIR (v10) [14] + PEATmoss (4902 stress-relevant orthogroups) [15], (2) eggNOG-mapper [16] (17 349 orthogroups) and (3) an intersection of both approaches (Wilcox test *p*‐value = 2.2 × 10^−16^), yielding 2475 orthogroups that are considered the final set of stress-relevant orthogroups. Presence of orthogroups in species is indicated by black (and absence by white).

Photosynthetic eukaryotes have a history of more than 1 billion years [1]. Many algal lineages have successfully made the wet-to-dry transition [2–4]. But only Embryophyta (land plants) rose above the substrate, evolved complex multicellular bodies and globally conquered land, constituting an evolutionary singularity [5–8]. Embryophytes belong to the Streptophyta, which encompass: (i) the Embryophyta (land plants), recovered by all molecular phylogenetic and phylogenomic analyses as a monophylum and united by synapomorphies that include the name-giving embryo [9], an alternation of generations [10] and likely the symbiotic interaction with mycorrhizal fungi [11,12]; and (ii) the paraphylum of streptophyte algae. Among the algae in this paraphylum, Charophyceae, Coleochaetophyceae and Zygnematophyceae are close relatives to land plants; together with land plants, they form a monophylum called Phragmoplastophyta (figure 1*a*). Therein, Zygnematophyceae are the closest algal relatives to land plants [17–19].

The independent wet-to-dry transitions in non-embryophytic plants incites the question: What was special about the biology of embryophytes that enabled them to dominate the land environment? There is a standing concept that the water- and/or land-dwelling streptophyte algal progenitor of land plants bore a mixture of exaptations and adaptations [6,7,20]. The genome sequences of extant members of the green lineage (Chloroplastida) provide a window through which to infer genetic potential in the ancestors along the trajectory of streptophyte evolution.

Comparative genomic analyses reveal that a substantial fraction of molecular physiological tools that land plants use to cope with terrestrial stressors can also be found in streptophyte algae, their last common ancestors (LCAs), and the LCA of streptophyte algae and land plants; all these ancestors had iterations of the complex genetic networks of land plants [21–27]. Several aspects have been investigated in the past decade through comparative analyses. One of the foremost are the deep evolutionary roots of phytohormone signalling cascades. For example, a complete chassis of homologues for the signalling cascade of the stress hormone abscisic acid (ABA) was found in several Zygnematophyceae [26,28,29] but functional analyses revealed that the protein homologues, albeit inhibiting downstream phosphatases, act ABA-independently [30]. Another interesting evolutionary pattern was recovered for the phytohormone auxin, whose polar distribution determines diverse aspects of growth and development in land plants [31–33]. Relevant homologues for the nuclear auxin response can be found in streptophyte algae [34–36] but a clear nuclear auxin response remains elusive in streptophyte algae. Where we do find a clear case of an auxin response conserved between land plants and streptophyte algae is the one that funnels through fast phosphorylation [37]. Next to phytohormones are several other specialized metabolites with important adaptive functions for terrestrial life, including phenylpropanoid-derived compounds [38], especially flavonoids [39] and unique algal compounds [40–42]. And of course, it has been pinpointed that the aforementioned adaptive symbiosis often requires genes to have had a deep evolutionary origin [11,43,44].

It has been proposed that the genes for these complex traits and networks arose in bursts of genetic novelty [45]; importantly, these burst are also recovered when corrected for homology detection failure [46]. But what made these bursts possible? Here, we explored the genetic substrate that underlies the complex stress-associated genetic networks now acting in land plants. From 37 species, we assembled a dataset of 57 795 orthogroups, extracted 2475 stress-associated orthogroups and traced their evolutionary assembly from protein domains. We infer that a quarter are land plant innovations, of which 15% emerged through novel domain shuffling. Our data explain that land plant-specific bursts of novelty for stress responses can arise from pre-existing protein domains, which are newly combined to create genetic novelty. New genes should allow for new network connections and fine-tuned responsiveness during the dawn of embryophytes.

## 2. Results and discussion

### (a) Assembling a genetic set for plant stress response

To trace the evolution of the elaborate molecular chassis for the response to stressors, we clustered all homologous proteins (OrthoFinder [47]) from predicted proteomes of five cyanobacteria, six chlorophyte algae, seven streptophyte algae and 19 land plants (figure 1*a*) into 57 795 orthogroups. Our dataset is highly diverse in terms of represented phylogenetic lineages, functional capacities and genome sizes. Species were selected to counterbalance existing biases in genome sequencing efforts by selecting representatives of all main branches in the green lineage. This species selection should cover a major share of the functional genomic diversity that is relevant for understanding the deep evolution of molecular functions of streptophytes. Thus, our analysis should not be influenced by the redundancy (or lack thereof) of protein families and variation of genome sizes. However, it is likely that additional genomic sequencing of species in key phylogenetic positions will in the future help to identify more complex evolutionary patterns. To identify stress-associated orthogroups, we used complementary approaches (figure 1*b*; §3 and electronic supplementary material, figure S1*a*,*b*), yielding 2475 orthogroups (figure 1*b*). While annotations here derive from land plants, the proteins often have deeper evolutionary origins. To scrutinize this, we searched for more distant homologues across the phylodiverse set of eukaryote proteins in the EukProt database [48]. The homology searches of these 2475 orthogroups include very few distant homologues in non-chloroplastidal species (electronic supplementary material, figure S1*c*); we thus focused on 32 Chloroplastida species. Our results suggest that while most stress response-related proteins occur throughout the green lineage (Chloroplastida), bursts in numbers of proteins per orthogroup with these domains have occurred at several points during evolution. We scrutinized this in the following.

### (b) Bursts in protein domains across the evolution of Chloroplastida

**Figure 2.**
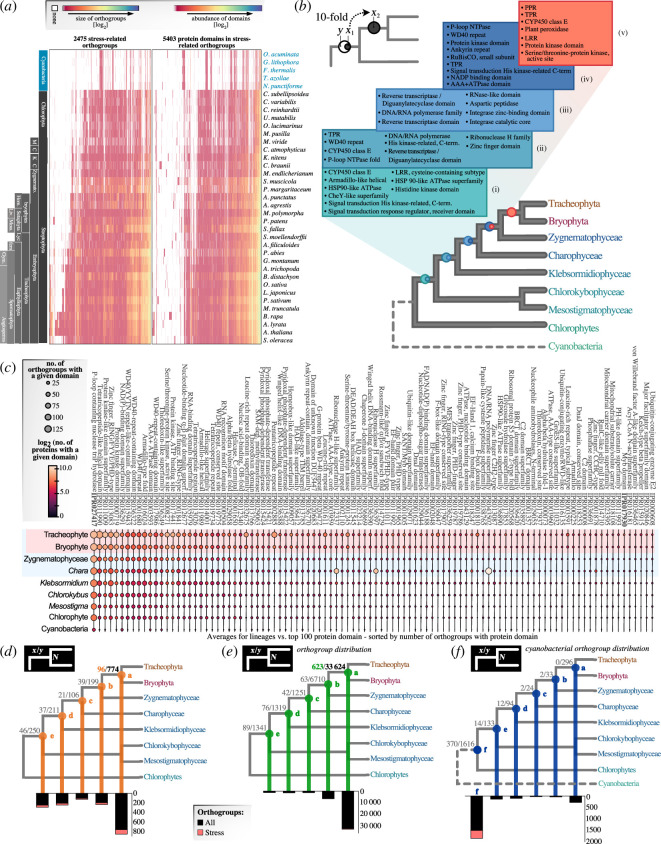
Evolution of stress-associated domains and orthogroups across streptophytes. (*a*) Heatmaps on the number of proteins in the 2475 orthogroups (left) and the abundance of 5403 protein domains from stress-relevant orthogroups (right; both measures are log_2_-transformed; absences are in white). The *x*-axes are sorted by hierarchical clustering. (*b*) Bursts in protein domain expansions across the backbone of the streptophyte phylogeny. Named protein domains in the coloured boxes are those that expanded by ≥10-fold between adjacent LCAs (coloured nodes). Bottom-up, the LCAs correspond to: (i) streptophytes, (ii) Phragmoplastophyta + Klebsormidiophyceae, (iii) Phragmoplastophyta, (iv) Zygnematophyceae + land plants, (v) land plants. (*c*) Frequency of protein domains across the orthogroups in the dataset, averaged and collapsed into major lineages (individual values can be found in the electronic supplementary material, figure S3). The top 100 most frequent protein domains (*x*-axis) are sorted by their frequency of occurrence in stress-relevant orthogroups. Bubble colours indicate the number of proteins in which a given domain occurs; values are log_2_-transformed (number of proteins) and plotted as a gradient from black (lowest), to purple, red and white (highest). Bubble size indicates the number of orthogroups that bear a given domain; background colours: land plants (red) and Phragmoplastophyta (blue). (***d–f***) Inference of the: first occurrence of domains in stress-relevant orthogroups and their assemblage into the domain order in extant species, analysing the contribution of orthogroups from previous lineage/s to LGP of the next lineages. The *x* in *x*/*y* represents the number of orthogroups at node *N* that have protein domains pertaining to the orthogroups (embryophytic domain) present at all previous nodes (*N* − 1, and *N* − 2, and *N* − 3, etc.) but that did not occur in a single protein in the same order before node *N*. The *y* in *x*/*y* represents the number of orthogroups at node *N* that have key protein domains pertaining to the orthogroups (embrophytic domain) immediately at *N* − 1. (***d***) Distribution of stress-annotated and overall orthogroups with LGP in terms of conserved embryophytic domain set across the successive LCAs (represented as *x*/*y*). (***e***) Distribution of any stress-annotated and overall orthogroups (*x* and *y* represented as *x*/*y*) across successive LCAs. (***f***) Distribution of cyanobacterial stress-annotated and overall orthogroups across the successive LCAs (similarly represented as *x*/*y*). Abbreviations: PPR = pentatricopeptide repeat; TPR = tetratricopeptide repeat; LRR = leucine-rich repeat; HAD = haloacid dehydrogenase; TIM = triose-phosphate isomerase; FYVE/PHD = **F**ab 1 (yeast orthologue of PIKfyve), **Y**OTB, **V**ac 1 (vesicle transport protein), and **E**EA1/plant homeodomain; SANT = **S**wi3, **A**da2, **N**-Cor, and **T**FIIIB; RING = Really Interesting New Gene; MFS = Major Facilitator Superfamily; BRCT = **Br**east Cancer Gene 1 **C t**erminus. Note that the DEAD/DEAH box family is named after the conserved Walker B motif (D-E-A-D sequence) and Myb after the Myeloblastosis genes.

To understand the emergence of a protein toolkit for stress response, we traced how these proteins emerged from domain building blocks. We used InterProScan [13] to predict the domain composition of all 57 234 proteins within 2475 orthogroups (figure 2*a*). We identified up to 5403 different domains (figure 2*a* and electronic supplementary material, figure S2). Most proteins are from tracheophytes, followed by bryophytes, streptophyte algae, chlorophyte algae and cyanobacteria (electronic supplementary material, figure S2). Yet, the majority of stress-associated orthogroups contained representatives across the green lineage, followed by tracheophyte-specific orthogroups and orthogroups shared across the entire dataset (i.e. also including cyanobacteria; electronic supplementary material, figure S2*b*). Among all 31 stress gene ontology (GO)-terms used as seed, most proteins were assigned to the category 'response to topologically incorrect protein' followed by 'cellular response to stress', both pertaining to common responses to various stresses. These were followed by several categories specifically related to environmental stresses. This captures well on a protein level that stress response derives from the requirement to maintain cell homeostasis.

From which protein domains were stress-relevant proteins built during evolution (figure 2*a*)? The two main mechanisms are the emergence of new domains and the reassembly of existing ones. To quantify this, we asked which biological functions are associated with bursts of domain emergence and changes in proteins’ domain architectures. Our focus was on protein domains that emerged in the LCAs of: (i) streptophytes, (ii) various streptophyte algal ancestors including (iii) Phragmoplastophyta, (iv) Zygnematophyceae + land plants, and (v) land plants. We measured the differential increments in protein domains from one ancestral tree node to the next by assessing the differences in the mean number of protein domains between the extant lineages that derived from the respective ancestral nodes, henceforth simply referred to as fold change. Here, we had a particular look at the top 10-fold changes from one ancestral node to the next (figure 2*b*). Particularly noteworthy were recurring expansions salient to: (i) gene editing in phragmoplastophytes (PPR, TPR) [49,50], (ii) CYP450 class E family within streptophytes, coherent with embryophyte chemodiversity [51,52] and (iii) signal transduction across streptophyte evolution.

### (c) Changes in domain usage recapitulate the evolution of signalling and regulation

An increase of proteomic complexity might follow two main scenarios: (i) an increase in the number of orthogroups with stress-associated domains, which suggests a diversified context for use of the same domains in different orthogroups; (ii) an increase in the number of proteins with stress-associated domains within orthogroups, which suggests duplications of genes coding for proteins with the stress-associated domain. A combination of (i) and (ii) can be most parsimoniously explained by sub- and neofunctionalization driven either by genetic divergence and/or domain-shuffling. Further, a less likely (non-parsimonious) scenario is duplication of just the domain and integration into another protein concomitant with the loss of the original orthogroup.

We quantified the contribution of 5403 protein domains to the 2475 stress-relevant orthogroups (figure 2*c*; electronic supplementary material, figures S3, S4; electronic supplementary material, table S3). While the 2475 orthogroups consist of proteins that are largely specific to the green lineage (electronic supplementary material, figure S1*c*) and therein particularly vascular plants, the protein domains are more ancient: the most frequent stress domains occurred either: (i) in a deep ancestor with prokaryotes or (ii) upon the emergence of the green lineage (figure 2*c*); none of the top contributors emerged exactly at the origin of either streptophytes or embryophytes. Some of the most pronounced changes occurred in the usage of domains salient to signalling, such as IPR000719 and IPR01109 (protein kinase(-like) domain) among the top five hits. Both are prominent across all lineages and showcase an increase in first abundance of proteins followed by orthogroup expansions upon the emergence of Phragmoplastophyta and of land plants (figure 2*c*). This speaks of a fine-tuning of environmental input and—the likely ancestral—multicellular growth of Phragmoplastophyta [26,53]. Indeed, among the top 100 changes in orthogroup numbers with stress domain-containing proteins, 90.2% of the relevant LCAs show a stronger increase in protein numbers than in orthogroup numbers. This highlights the propensity of gene duplications in gene families salient to stress responses, similar to morphogenic processes [54].

Next to small-scale duplications, we recover several bursts ascribed to major streptophyte lineages (see more on this below in the following paragraphs). The most used domain in stress-relevant orthogroups in land plants was IPR027417 (P-loop containing nuclease triphosphate hydrolase), with 27 379 occurrences in the 19 land plant genomes. The most common domain exclusive to land plants is IPR002902 (Gnk2-homologous domain), with 4359 occurrences; the superfamily of proteins with an Gnk2-homologous domain is highly diversified and associated with the protection of the seed from biotic and abiotic environmental threats [55,56].

**Figure 3.**
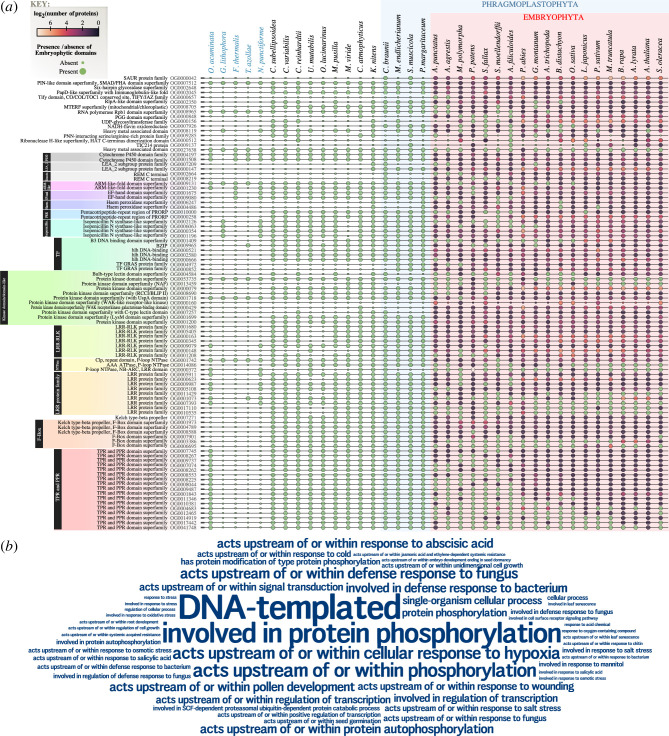
Latent genetic potential (LGP) in the protein building blocks for plant terrestrialization. (*a*) Phylogenetic distribution of key protein domains contributing to 96 stress-relevant orthogroups described in land plants. The bubble plot shows: (*i*) the 37 species/datasets on the *x*-axis; (ii) the 96 orthogroups and their corresponding functional annotations on the *y*-axis; (iii) presence (big circle) or absence (small circle) of embryophytic domains pertaining each orthogroup, and (iv) the count (log_2_-transformed) of proteins within each orthogroup, from black (absent) to white (highest). (*b*) Word cloud showing the top 50 most frequent Gene Ontology (GO) annotations (‘Biological Function’ via eggNOG-mapper) for proteins from the 96 orthogroups at the LCA of land plants node (node a in figure 2*d*). Word size represents GO term frequency.

In addition to the above observed pattern, some domains showed an increase in the number of proteins yet only small or no effects on the number of orthogroups (figure 2*c*). It is conceivable that an increase of proteins within the same orthogroup reflects less-divergent gene duplicates that are subfunctionalized, whereas an increase of proteins and of orthogroups indicates higher sequence divergence or domain composition, speaking of neofunctionalization. Yet, it is difficult to use protein and orthogroup patterns to derive biological functions, which are anyway defined loosely and not directly comparable among gene families. It is nonetheless interesting to speculate that under neofunctionalization we can expect more orthogroups, whereas under subfunctionalization the duplicates often will be clustered within the same original orthogroups and thus without an increase in orthogroup numbers. Examples include regulatory and signalling protein domains such as the transcription factor-associated Myb domain (IPR017930; figure 2*c* and electronic supplementary material, figure S3) and the signal transduction domains of the CheY-like superfamily (IPR011006; figure 2*b*). A similar pattern is observed with conserved domain (‘site’) of CYP450 (IPR017972; figure 2*b*), corroborating the high degree of subfunctionalization and metabolic versatility observed in enzyme families with these domains [57], and the ABC transporter-like ATP binding domain (IPR003439; figure 2*b*) and ATPase AAA core (IPR003959) domains (figure 2*b* and figure 3*a*). Some changes recapitulate signature expansions, e.g. PPRs [58], with IPR002885 (PPR) having undergone an increase of protein domains in land plants by a fold change of 2000 (i.e. the difference between the average number of IPR002885 domains in the LCA of land plants and the LCA of Phragmoplastophyta) (figure 2*b,c*). Similarly, leucine-rich repeat (LRR) domains IPR032675 and IPR003591 both increased upon the emergence of seed plants (electronic supplementary material, figure S3), concomitantly with a burst of nucleotide-binding site–leucine-rich repeat (NBS-LRR) resistance genes [59]—a burst that brought forth a new regulatory mechanism via microRNA family miR482/2118, regulating both stress responses and reproductive development, originating first in the LCA of seed plants [59,60].

Hundreds of F-box proteins occur in vascular plant genomes, where they recruit specific substrates to ubiquitin proteasome-based regulation [61]. We observed an increase of F-box protein family domains (IPR036047 and IPR001810; figure 2*c* and electronic supplementary material, figure S3) in land plants, and more so in angiosperms. F-Box proteins have apparently diversified in tracheophytes; concomitantly, both domains are reduced in liverworts and mosses, speaking to a combination of multiple events of duplication among tracheophytes and the reductive sweep in bryophyte genome evolution [62]. A similar trend occurs in the protein kinase domain (IPR000719; figure 2*c* and electronic supplementary material, figure S3) and the serine/threonine protein kinase domain (‘active site’; IPR008271; figure 2*c* and electronic supplementary material, figure S3). Taken together, we conclude from our data that domains integral to high-level signalling processes were seeded before plant terrestrialization but actualized through (often bursts in) usage of these domains in novel biological commitments.

### (d) Genetic potential gleaned from protein domain emergence and assembly

The first land plants bore adaptations and exaptations to alleviate the stresses of the terrestrial habitat [6,7,20]. Exaptations are traits co-opted for a new function that either had a different ancestral (adaptive) function or were non-adaptive [63]. At the molecular level, protein domains represent the raw material from which new proteins can arise through new domain combinations. By integrating domains into new contexts—forming new protein architecture—or by being expressed in a different biological program, novelty in form and function can arise. This speaks of latent genetic potential (LGP) in the ancestors of land plants for assembling molecular programmes to respond to terrestrial stressors. Some of this should bear out from our data. We therefore explored the evolutionary time lapses between the emergence of *bona fide* stress-related proteins and the deeper origins of their individual protein domains.

Of the 2475 orthogroups, 623 had representatives in at least one angiosperm and one bryophyte, but in no other lineage outside of land plants (figure 2*e*), thus emerging in the LCA of land plants. Hence, one-quarter of stress-related orthogroups emerged with land plants. We determined the LCAs in which each protein domain first occurred for all 2475 stress-associated orthogroups (figure 2*d*).

We focused on the emergence of the 2475 stress-annotated orthogroups (*x*) and the total 57 795 orthogroups (*y*) (for comparison, see *x*/*y* representations in figure 2*e*). Here, our data capture the genetic bursts in the LCAs: (i) of land plants and Zygnematophyceae and (ii) of land plants (figure 2*e*, nodes *a* and *b*). Chloroplastida and cyanobacterial proteins are included in 2196 orthogroups, potentially capturing a signal from endosymbiotic gene transfers from plastids to nuclear genomes [64] (figure 2*f*). Out of these 2196, 400 are annotated as stress-related. Our data suggest that none of them contributed to stress-related genetic novelties that emerged with embryophytes, but instead were deployed earlier during evolution. We further validated this effect by searching for cyanobacterial homologues in the EukProt dataset (electronic supplementary material, table S1).

Do stress-annotated orthogroups emerge de novo or by combining pre-existing protein domains? We investigated how many land plant-specific orthogroups (figure 2*d*) are formed by domains with older evolutionary origins (figure 3*a*). For example, Remorin (OG0002664 and OG0008219; for all orthogroups, see electronic supplementary material, table S2) appear exclusively in embryophytes (figure 3*a*) but Remorin domains (‘C terminus’) are also present in *Chara braunii*, and thus they were likely present in their LCA. In total, 96 out of 623 (about 15%) are stress-annotated compared to 774 out of 33 624 (about 2%) overall embryophyte-specific novelty emerging from LGP (compare figure 2*d*,*e*).

Based on the most frequently predicted functions within these 96 orthogroups, we infer that the genetic potential for birth and expansion of homologues could have aided in signalling, molecular transport, microbe interaction, phenolic polymer formation, transcriptional regulation and RNA editing during terrestrialization (figure 3*b*). The embryophytic domains for JAZ proteins (OG0000657) occur across streptophyte algae, suggesting that its domains were present in the streptophyte LCAs. That said, JAZ proteins (OG0000657; electronic supplementary material, figure S3) belong to the 96 embryophyte-specific proteins, aligning with previous data indicating that functional JAZ and the co-occurrence of all the required domains are limited to embryophytes [65,66]. Yet, our data show that the LGP for a key component of the jasmonate signalling pathway, JAZ, was dwelling already in streptophyte algal ancestors and that functional JAZ derive from reshuffling and/or new domain combinations.

Streptophyte algae have orthologues for the genetic toolkit that in land plants establishes interactions with symbiotic fungi [43], especially genes involved in upstream signalling processes. Further, *C. braunii* has an expanded repertoire of LysM receptors [67]. Our data captured the RLK and LysMs orthogroups and proteins homologous to CERK1 in OG0001699 (electronic supplementary material, figure S3) and show that this orthogroup appeared in the LCA of land plants. Here, too, all its domains—the embryophytic domain assembly—were already present before the LCA of Chloroplastida. While chitin recognition via CERK1 homologues likely first emerged in the LCA of land plants [68], the LGP (i.e. its building blocks) is ancient.

Evolution of amino acid sequences in domains has shaped the functions that they fulfil in the genetic, protein and cellular context in which they reside. Thus, certain domain variants can have such divergent sequences that they are categorized as different domains. This has consequences for the insights garnered here. Identified changes can refer to the appearance of a new domain or a new domain variant. Thus, it is likely that certain domains are even more ancient than we currently estimate here. Overall, we suggest that the embryophyte-specific stress response toolkit (96 orthogroups) has a much earlier evolutionary origin and radiation if we consider the deep origin of the protein domains (figures 2*c*,*d* and 3*a*).

### (e) Conclusion

Extant streptophytes display a mosaic of traits, brought about by processes like parallelism, convergence and rampant gene loss of key traits, concomitant with genomic streamlining in the Zygnematophyceae [20,26,62,69,70]. Yet, it is difficult to infer deep evolutionary processes that underlie genomic novelty by looking only at extant species: properties salient to a terrestrial lifestyle shared by land plants and streptophyte algae can be interpreted as exaptations [6], or adaptations under the assumption that the LCA of streptophytes was a terrestrial organism [71]—but there have been multiple habitat transitions [72]. Here, we reconciled trait mosaicism, bursts in genetic novelty and the deep evolutionary origins of protein domains underpinning stress response in land plants. We propose that domain co-option driven by integrations into new biological contexts was an important force in streptophyte evolution: in our analyses, domain co-option explains at least 15% of the apparent genetic novelty in land plants—likely an underestimate given the conservative nature of our analyses. Molecular traits emerging from LGP include the moulding of hallmark signalling, biotic interactions and specialized metabolic processes. This is likely no coincidence, consistent with signalling network remodelling [26,73], biotic stress-driven co-evolution [74] and the promiscuity of specialized metabolism [75], and as such a hotbed for biological novelty. Overall, our data suggest that novel combinations of old genomic substrate shaped the plant terrestrialization toolkit.

## 3. Methods

### (a) Assembly of a protein database across the green lineage

We downloaded predicted proteins from genomes of: (i) cholorophyte algae [76–81], (ii) streptophyte algae [22,29,39,67,82] (following naming [83]), (iii) bryophytes [84–87], and (iv) tracheophytes [14,88–101]. Additionally, Cyanobacteria were included [102–106]. Completeness was assessed using BUSCO v. 4.1.4 [107] with the ‘Viridiplantae odb10’ dataset (parameters: e-value threshold 0.001, three candidate regions considered per BUSCO).

### (b) Stress-annotation of protein data

We used OrthoFinder2 [47] on all proteomes and obtained 57 795 orthogroups (full analysis mode; default parameters). We searched for stress-related proteins in either *Arabidopsis thaliana* [14] or *Physcomitrella patens* PEATmoss [15] and used these proteins to annotate orthogroups. For *A. thaliana*, we extracted stress-relevant proteins based on 31 parent Gene Ontology (GO) terms (GO:0001666, GO:0002931, GO:0003299, GO:0006952, GO:0006970, GO:0006979, GO:0006991, GO:0009271, GO:0009408, GO:0009409, GO:0009413, GO:0009414, GO:0009611, GO:0009635, GO:0033554, GO:0033555, GO:0034059, GO:0034405, GO:0035900, GO:0035902, GO:0035966, GO:0042594, GO:0051409, GO:0051599, GO:0055093, GO:0061771, GO:0080134, GO:0090664, GO:0097501, GO:0097532, GO:1990911) and all their children (yielding 11 641 Arabidopsis proteins). For *P. patens,* we worked with the GO annotations provided by PEATmoss [15]. Among the total of 57 795 orthogroups, we identified 4902 as being relevant to ‘stress’ whenever they contained at least one stress-relevant protein each from *Arabidopsis* and *P. patens*. Independently, we performed de novo GO term assignment of orthogroups using eggNOG-mapper (*e*-value threshold 0.001, minimum bit score 60, 1 best HMM-hit reported, sequences >5000 bp ignored, database size 40 000 000 and diamond v. 0.9.24). This approach yielded 17 349 stress-related orthogroups after parsing de novo annotations for the occurrence of at least one of the aforementioned 31 GO terms or its children.

We intersected the two annotation methods, yielding 2475 stress-annotated orthogroups out of the initial 57 795 orthogroups see electronic supplementary material, figure S2 for details).

### (c) Stress-annotated orthogroup distribution across EukProt

Homology of proteins from 2475 stress-annotated orthogroups was checked against the EukProt database using DIAMOND v. 0.9.24 [108,109] with *e*-value <1 × 10^−22^ and percentage identity as >25%. The EukProt database comprises annotated proteins from 993 species across eukaryotes, out of which 118 species belong to Chloroplastida; the remaining 875 belong to Glaucophyta, Rhodophyta, TSAR, Cryptista, Haptista, Amorphea, Metamonada, Discoba, CRuMs, Hemimastigophora, Malawimonadida and Ancyromonadida.

### (d) Domain prediction

Protein domains were predicted for 37 proteomes using InterProScan 5 [13] using all databases (CDD + COILS + Gene3D + HAMAP + MOBIDB + PANTHER + Pfam + PIRSF + PRINTS + PROSITE + SFLD + SMART + SUPERFAMILY + NCBIFAM; parameters: default pre-calculated match lookup service using MD5 checksum, *n* = 8 longest ORFs taken as input for analysis). Protein domains predicted totalled 14 367, out of which 5403 were found in the 2475 stress-relevant orthogroups. To compute protein domain frequencies, the intervals of occurrences were merged given that the domain is predicted more than once on overlapping intervals. The count and domain order of proteins are finalized based on merged intervals and the total frequency of protein domain occurrence in all species and orthogroups is computed.

For fold changes in protein domains predicted in the 2475 stress-annotated orthogroups between sister lineages, the average count of a given domain in the LCA of a lineage was divided by the average count in its sister lineage. The top 10-fold changes of protein domains between adjacent LCAs (nodes) are reported in figure 2*b*.

### (e) Embryophytic domain set and latent genetic potential

We define ‘embryophytic domain set’ as the domain configuration in a given orthogroup that originated in the land plant LCA, i.e. each of the domains in the set is predicted in at least one species of: (i) Tracheophyta and (ii) Bryophyta. For a given species, we define domain completion when all the domains in the embryophytic domain set are predicted for this species. We calculated (*x*/*y*) of orthogroups at each node (a–f). For example, the numbers at node b indicate the count of orthogroups in Tracheophyta + Bryophyta + Zygnemaotphyceae that have LGP (or key Embryophytic protein domains) in all the rest of the lineages (Charophyceae + Klebsormidiophyceae + Chlorokybophyceae + Mesostigmatophyceae + Chlorophytes). We define LGP as the case when the domains that make up a given protein were individually present at an earlier evolutionary time; thus, the required domains emerged earlier than the orthogroup. The origin of orthogroups is assessed parsimoniously assuming common ancestry of all the represented taxa.

### (f) Data visualization

The heatmap plot was created using gplots (https://cran.r-project.org/web/packages/gplots), curl (https://cran.r-project.org/web/packages/curl), dendextend [110], colorspace (https://cran.r-project.org/web/packages/colorspace/index.html) and RColorBrewer (https://cran.r-project.org/web/packages/RColorBrewer). We used ggplot2 [111], viridis [112] and reshape2 [113] for bubble plots. For the respective R packages, R v. 4.0.3 [114] was used. Upset plots were created using pandas, matplotlib.pyplot and upsetplot packages in Python v. 3.9.5. The wordle was created using wordle.net.

## Data Availability

Code is available online [115]. All computational analyses were performed with published tools and are cited in §3. All intermediate files were deposited on Dryad [116]. Supplementary material is available online [117].
